# Environmental factors influence cross-talk between a heat shock protein and an oxidative stress protein modification in the lizard *Gallotia galloti*

**DOI:** 10.1371/journal.pone.0300111

**Published:** 2024-03-12

**Authors:** Edward Gilbert, Anamarija Žagar, Marta López-Darias, Rodrigo Megía-Palma, Karen A. Lister, Max Dolton Jones, Miguel A. Carretero, Nina Serén, Pedro Beltran-Alvarez, Katharina C. Wollenberg Valero

**Affiliations:** 1 School of Natural Sciences, The University of Hull, Hull, United Kingdom; 2 Energy and Environment Institute, The University of Hull, Hull, United Kingdom; 3 National Institute of Biology, Ljubljana, Slovenia; 4 CIBIO Research Centre in Biodiversity and Genetic Resources, InBIO, Universidade do Porto Campus de Vairão, Vairão, Portugal; 5 Instituto de Productos Naturales y Agrobiología (IPNA-CSIC), La Laguna, Tenerife, Canary Islands, Spain; 6 Universidad de Alcalá (UAH), Biomedicine and Biotechnology, Alcalá de Henares, Madrid, Spain; 7 BIOPOLIS Program in Genomics, Biodiversity and Land Planning, CIBIO, Campus de Vairão, Vairão, Portugal; 8 Biomedical Institute for Multimorbidity, Centre for Biomedicine, Hull York Medical School, The University of Hull, Hull, United Kingdom; 9 Department of Fish and Wildlife Conservation, Virginia Tech, Blacksburg, VA, United States of America; 10 Departamento de Biologia, Faculdade de Ciências, Universidade do Porto, Porto, Portugal; 11 School of Biology and Environmental Science, University College Dublin, Belfield Campus, Dublin, Ireland; Oklahoma State University, UNITED STATES

## Abstract

Better understanding how organisms respond to their abiotic environment, especially at the biochemical level, is critical in predicting population trajectories under climate change. In this study, we measured constitutive stress biomarkers and protein post-translational modifications associated with oxidative stress in *Gallotia galloti*, an insular lizard species inhabiting highly heterogeneous environments on Tenerife. Tenerife is a small volcanic island in a relatively isolated archipelago off the West coast of Africa. We found that expression of GRP94, a molecular chaperone protein, and levels of protein carbonylation, a marker of cellular stress, change across different environments, depending on solar radiation-related variables and topology. Here, we report in a wild animal population, cross-talk between the baseline levels of the heat shock protein-like GRP94 and oxidative damage (protein carbonylation), which are influenced by a range of available temperatures, quantified through modelled operative temperature. This suggests a dynamic trade-off between cellular homeostasis and oxidative damage in lizards adapted to this thermally and topologically heterogeneous environment.

## 1. Introduction

The molecular mechanisms underlying environmental adaptation are complex and constrained by fundamental biochemistry, especially concerning thermal adaptation and water balance [1]. Genes involved with the expression of physiological traits relating to environmental change can be differentially regulated in response to periods of cellular stress [2], and so can constitutive (baseline) protein expression levels [3], which can reach optima through evolutionary adaptation [4–6]. Amongst these mechanisms, are “frontloading” [7, 8] or “preparation for oxidative stress” [9, 10], which act to physiologically prepare organisms for frequently encountered environmental stressors. This reduces the requirement for an inducible stress response [7, 11], and links plastic responses to evolutionary processes [2, 10]. In the context of climate change, more rapid plastic responses are vital for survival, with extreme weather events happening outside of evolutionary time-scales [12]. Heat tolerance has been linked to adaptive evolution in populations of *Anolis* lizards via differentially expressed genes [13], demonstrating that the frequency of stressors can lead to environment-mediated selection at phenotypic, genomic, and regulatory levels.

Of relevance to environmental optima and cellular stress, heat-shock proteins (HSPs) play a vital role, protecting proteins from denaturation, misfolding, and aggregation [14]. HSPs occur both at constitutive levels as housekeeping molecular chaperones, and can be induced during stressful events [15], 2–6 hours after stressor exposure [16] and depending on stressor duration [6, 17]. While they are regarded as important biomarkers for responses to acute thermal and other environmental stressors, studies outside of laboratory conditions are less common [6, 18] and laboratory studies may only incompletely reflect conditions experienced in nature [19]. A member of the Heat Shock protein family, relevant to environmental settings, is GRP94 (Heat Shock Protein 90 Beta Family Member 1, also called endoplasmin, GP96, or TRA1, and coded by the *HSP90B1* gene). GRP94 is a glucose-regulated protein specifically found in the endoplasmic reticulum (ER) of cells, where it acts as a molecular chaperone folding secretory proteins and regulating protein-protein interactions [20]. These functions are vital to ER quality control pathways, with GRP94 expression linked to ER stress (leading to a global unfolded protein response) and to cellular immune response, amongst others. This makes GRP94 a relevant marker for whole-organism physiological stress [20, 21]. The expression of GRP94 has also been linked to regulating oxidative stress and protein carbonylation in cell lines and tissue models in the laboratory [22, 23]. Oxidative stress occurs during conditions in which oxidative load outweighs antioxidative mechanisms, potentially leading to cell damage and death [24]. Besides genetic and disease-related causes, oxidative stress can be caused by environmental factors and induced by several mechanisms, including pollutants, different forms of radiation, nutrition, and other environmental interactions [25]. Oxidative stress markers include, for example protein post-translational modifications (PTMs) such as 3-nitrotyrosine (3-NT) [26, 27] and carbonylation [28–30]. Some evidence exists for cross-talk between protective molecular chaperone activity and oxidative stress markers, where HSPs and HSP-like molecules play a role in protection against oxidative damage [31–33] and this evidence extends to natural populations (e.g., HSP70, HSP60, HSP90, HSC70, GRP75) [34].

Squamate reptiles are vulnerable to climate change, with extinction rates accelerating [35–39], and their capacity to adapt remains unclear [40–43]. Among these, lizards provided an early model for linking behavioural thermoregulation to physiology and performance [44–46] and remain relevant as a model to investigate adaptation to different environmental conditions at the molecular level [47]. For example, to link ectotherm thermoregulation to the environmental thermal profile, the operative temperature (T_e_) can be calculated, which estimates a steady-state body temperature based on available microclimates according to a biophysical model [48]. By calculating a T_e_, a more accurate picture of thermal tolerance limits to thermal stressors can be estimated, which can exceed environmental temperatures [49]. If operative temperatures can exceed physiological safety-margins, and can additionally be influenced by other stressors such as dehydration [50], then behavioural thermoregulation opportunities influenced by the microclimate are vital for the survival of small ectotherms such as lizards.

In ectotherms, behavioural thermoregulation can act to shield physiological traits from selection [42]; however, behavioural thermoregulation does not come without costs [51–54]. For example, hard physiological boundaries may constrain adaptation beyond certain thermal limits, which are not modulated by natural selection [55, 56]. An animal’s thermal sensitivities involve behavioural traits associated with reaching optimal performance, and physiological traits influencing thermal resilience. These are often accompanied by trade-offs, like to fitness [57–59], and are subject to differential evolvability [60]. However, the molecular basis of how adaptation to environmental optima is achieved, is less explored [47]. *Gallotia galloti* is a basal lacertid lizard endemic to the Canary Islands (Spain). In Tenerife it is abundant, and occupies all types of habitats, from the endemic coastal scrub up to the high mountain vegetation, including the top of El Teide stratovolcano, at3,715 m a.s.l. The species inhabits remarkably diverse environments, including varying thermal and hydric parameters, making this species an ideal model to study environmental adaptation [61, 62].

The macrohabitat of Tenerife comprises four main bioclimatic zones, ranging from hot and dry (Env A) at low elevations (average monthly temperature range 17.7–23.6°C, annual rainfall ~100 mm), to wetter and cooler mid-elevations further inland in the South (Env B) (average monthly temperature range 9.7–15.6°C, annual rainfall ~105 mm). In the Northern side of the island, slopes are characterised by higher rainfall and humidity (Env C) (average monthly temperature range 17.8–23.8°C, annual rainfall ~128 mm). The highest elevations around Teide experience highly variable temperatures and precipitation patterns (Env D) (average monthly temperature range 6.7–14.3°C) [61]. Further, contrasting microclimates within and between these environmental zones are vitally important in lizard ecology [63–65]. Diversification across Tenerife resulted in two subspecies with contrasting phenotype, *G*. *g*. *galloti* in the South slopes of the island and *G*. *g*. *eisentrauti* in the Northern slopes. However, two phylogenetic clades [66, 67] bisect each of these two subspecies into a North-Eastern and a South-Western lineage.Laboratory experiments indicate that preferred temperature remains similar across altitudes and that water loss is size-related within phylogenetic lineages, but that *G*.*g*. *eisentrauti* selects lower temperatures and has higher evaporative water loss than *G*. *g*. *galloti* [68]. This suggests a combination of ecophysiological conservativeness (and, hence, phenotypic plasticity) at shallow evolutionary levels and selection on both thermal and hydric traits at deeper evolutionary levels [68].

This study aimed to establish baseline expression of heat shock proteins GRP94 and HSP70, and levels of protein carbonylation and 3-NT modifications, indicative of oxidative stress, in both *G*. *galloti* subspecies across contrasting environments and microclimates on Tenerife. We hypothesised that: 1) higher levels of constituent chaperones (HSP70 and GRP94) are present in tail tips of *G*. *galloti* at higher altitude (with associated greater radiation, and lower temperature and humidity) or coastal lowlands (due to hotter temperatures); and 2) changes in the expression of chaperones correlate with changes in the levels of oxidative stress biomarkers (carbonylation and 3-NT). Specifically, we explore cross-talk between GRP94 and carbonylation, and if proxies of thermoregulatory ability (T_e_) align with biomarker expression.

## 2. Materials & methods

### 2.1 Tissue sampling

**Fig 1:**
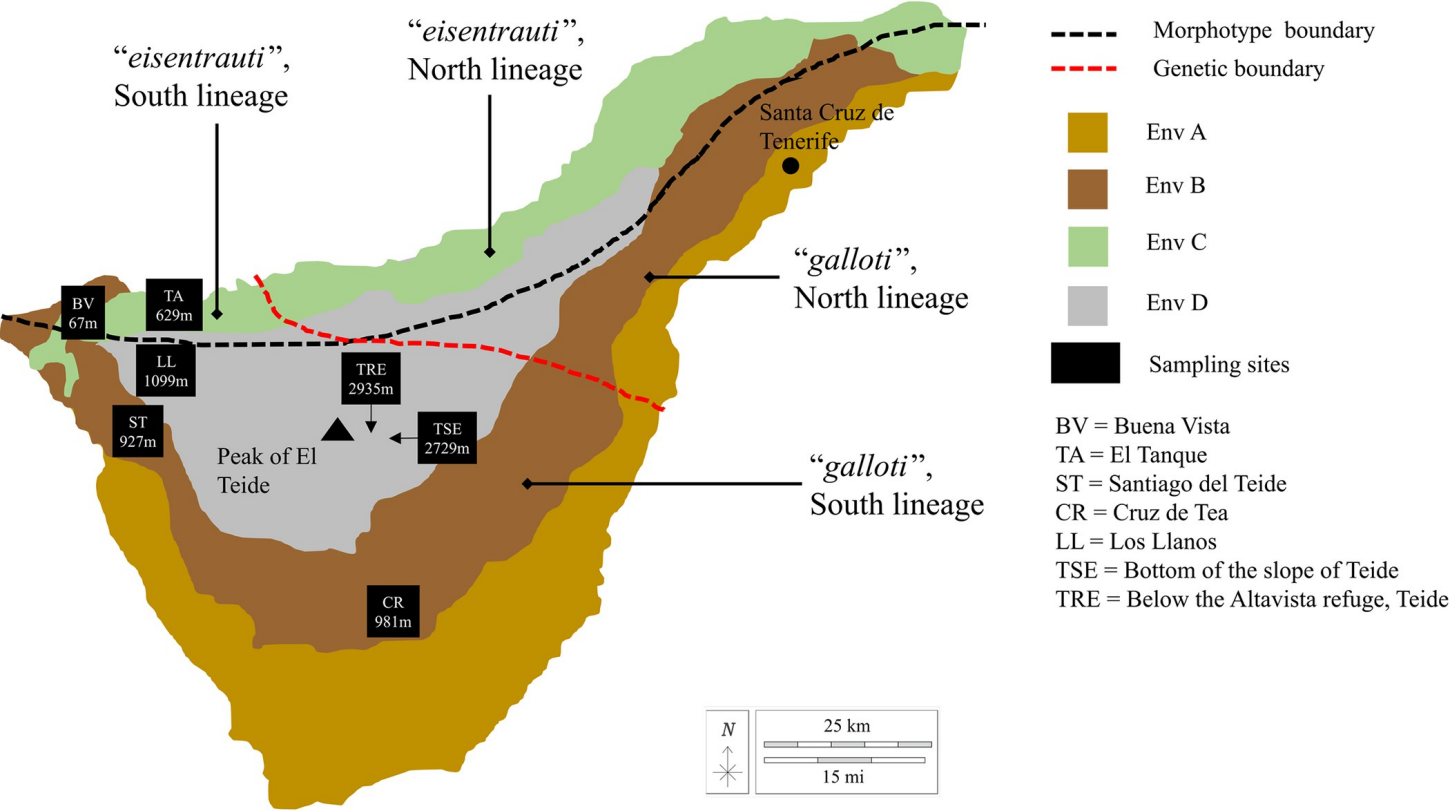
Map showing the approximate separation of the two morphotypes (subspecies) on the island, and approximate genetic separation, with labels showing the expected morphotype and clade in each area, based on Brown et al. [67]. Environmental zoning approximated from Algar & López‐Darias [61], showing the different environment types quantified on Tenerife. Environment C (green) occupies a similar distribution as the "eisentrauti" morphotype. Black squares indicate sampling sites with elevation in metres above sea level. Map outline and landmark features “El Teide” and “Santa Cruz de Tenerife” were traced from arcGIS (Esri, “Topographic” [basemap]. “World Topographic Map”).

Ethics approval was obtained for the study of preserved lizard tissues by the University of Hull (FEC_2022_17) and for fieldwork tissue collection from the Cabildo Insular de Tenerife and the Teide National Park (AFF 160/18 (2018–02258) and MDV/gap (28860)). Tail tips from *Gallotia galloti* were acquired from seven localities across the island of Tenerife during July 2018, covering various elevations and bioclimates (excluding Env A in Fig 1), and both subspecies (12 from *G*. *g*. *eisentrauti* and 16 from *G*. *g*. *galloti*), from just the “South” phylogenetic lineage (Fig 1). The tail tips consisted of scales, skin, muscle, blood vessels, and some bone and cartilage. Four biological replicates were obtained from each locality to yield 28 samples. Since these samples were involved in other research [68], we could not ensure balanced design of males and females, with the two highest-elevation populations sampled from females only. However, to account for this factor, sex was included as a predictor in statistical analysis. Tissues were snap frozen and stored at -80°C, and time-to-processing was included as another factor in statistical analysis (see S3 and S4 Tables in S1 File).

### 2.2 Protein extraction and antibody testing

Proteins were extracted from tail tip tissue, quantified with a Pierce BCA protein assay (ThermoFisher Scientific, Waltham, USA, 23225), and all samples were then normalised to a protein concentration of 1 μg/μl. We first tested the performance of antibodies against GRP94 and HSP70 for species cross-reactivity in reptiles, on a protein lysate from the gecko species *Hemidactylus turcicus*, and used this same lysate as a positive control for standardisation purposes on all membranes. Western blotting was then performed on all samples (detailed Methods are given in Supplementary Material 1 in S1 File).

To detect carbonylation, an Abcam Protein Carbonyl Assay Kit (Western blot, Cambridge, UK, ab178020) was used on new membranes for each set of biological replicates, with *H*. *turcicus* and negative controls. The manufacturer’s instructions were followed for derivation of the protein mixture plus controls, and samples were loaded, ran through SDS-PAGE, and transferred as above. Membranes were incubated with DNP primary antibody and HRP conjugated secondary antibodies included in the kit and visualised as above. To detect 3-NT, the above steps were followed except that a PVDF membrane activated in methanol was used and blocked using 5% BSA/TBST. The membrane was incubated with 3-NT antibody in 5% BSA/TBST at 1:1,000 dilution and signals visualised as above.

### 2.3 Band densitometry and standardisation

First, bands for each biomarker from each western blot image, as well as the associated total protein density (from Ponceau staining), were measured in ImageJ [69] for each lane. To standardise bands, a lane standardisation factor (see Supplementary Methods in S1 File) was calculated by dividing the lane value over the highest lane value on that membrane [70]. Biomarker band density was then divided by that lane standardisation factor. All bands present for the two candidate proteins, (GRP94 and HSP70) were quantified. Protein carbonylation was investigated at 250, 130, and 120 kDa bands, as well as the sum of these bands (described as “total carbonylation”). 3-NT bands were analysed at approximately 65, 55, 30, and 18 kDa, as well as the sum of these bands (described as “total nitrated tyrosine”).

### 2.4 Microclimate data

To obtain microclimatic parameters as predictors for biomarker expression, microclimate values at the scale of the individual lizard at each locality were modelled using *NicheMapR* and *microclima* packages in R [71], specifically using the *micro_*ncep function of the *NicheMapR* v.3.2.1 [72] R package. A raster object of site-specific elevation was accessed via the *get_dem* function of the *microclima* v.0.1.0 R package [71], which was used to generate microclimate temperature surfaces at 3 cm above ground (hgt = 0.03) using the *run_auto* function. 3 cm was chosen as a biologically relevant height given the size, vagility, and behaviours of *G*. *galloti* [72]. Modelled microclimatic variables at each locality are shown in S5 Fig in S1 File. Microclimate variables are adjusted when modelled, which represent different patterns compared with other bioclimate layers (e.g. solar radiation).

Microclimate temperature for each site was modelled separately for each locality on the date of collection, with mean, maximum, and minimum daily temperature generated from the temperature layers. The investigated biomarkers change in the timeframe of hours [16] to no more than a few days [73, 74]. We did not detect any sudden changes in environmental parameters before the sampling day (see S3, S4 Tables in S1 File), providing additional justification for modelling microclimate on the day of collection. Furthermore, the cardinal aspect and the slope angle for each site were extracted from a DEM layer of Tenerife [75] using ArcGIS pro (Version 2.8.0, ESRI).

### 2.5 Lizard hourly operative temperature

To put our results in the context of lizard thermal ecology, we modelled the hourly operative temperature (T_e_) of lizards at each locality using the *TrenchR* package [76] in R. T_e_ estimates a steady-state body temperature based on available microclimates according to a biophysical model [48].

To consolidate factors related to solar radiation and topology into a biologically relevant metric, and to investigate thermoregulatory opportunity for *G*. *galloti*, we modelled mean (over 24 h) T_e_ and investigated its relationship with GRP94 expression and total protein carbonylation. Variables used for the computation of T_e_ included: longitude and latitude, elevation, minimum and maximum air and soil temperature, wind speed, body temperature assumption (35.75°C, [77], date, average mass and average snout vent length, with all other values calculated in the package, using the model parameters set by [78].

### 2.6 Statistical analysis

In addition to the individual-scale microclimate data, additional predictor variables considered for statistical analysis were sex, elevation, subspecies, population ID, macroenvironmental category and sample processing time from field to tissue collection. To prevent model overfitting and statistical and computational viability, all candidate predictor variables were then checked for collinearity using the R package *corrplot* [79], and only one of two correlated variables greater than a Pearson’s correlation coefficient threshold of 0.9 were included (see S2 and S3 Figs in S1 File for variable selection). Final variables included in statistical analysis were slope angle, sex, morphotype (subspecies), elevation, population, daily average temperature, environment category, relative humidity, soil surface wetness, solar radiation, and sky radiant temperature.

In order to test the effect of these 11 variables on the expression of biomarkers, the R package *glmulti* [80] was first used to determine the best explanatory model for each biomarker as follows: Lowest Akaike Information Criterion (AICc) score was used as an initial selection criterion, firstly running an exhaustive algorithm for each biomarker without interactions, and then repeating the search with only variables in the top selected models from the exhaustive search (ΔAICc = 2), and the top four model-averaged terms. Model selection was then re-run allowing for interactions between predictors using the selected terms, and the final model was selected based on inclusion of variables in the top selected models within a ΔAICc of 2 [81], and those occurring within the top ranked model-averaged predictor terms [82].

Due to non-normality of the biomarker response variables, generalised linear models (GLZ) were run with the best model for each biomarker followed by pairwise Wilcoxon tests in R. Significant variables included in the final models were visualised using the *interactions* package [83], and *post-hoc* estimated marginal means (*emmeans*), [84] were calculated for any predictor variables included in each best model. We also explored the relationship between biomarker expression across locality, between proteins and PTMs, and between mean T_e_ and biomarker expression through Spearman’s rank correlations and Kruskal-Wallis tests.

## 3. Results

### 3.1 Expression levels of GRP94, but not of HSP70, vary with environmental factors

**Fig 2:**
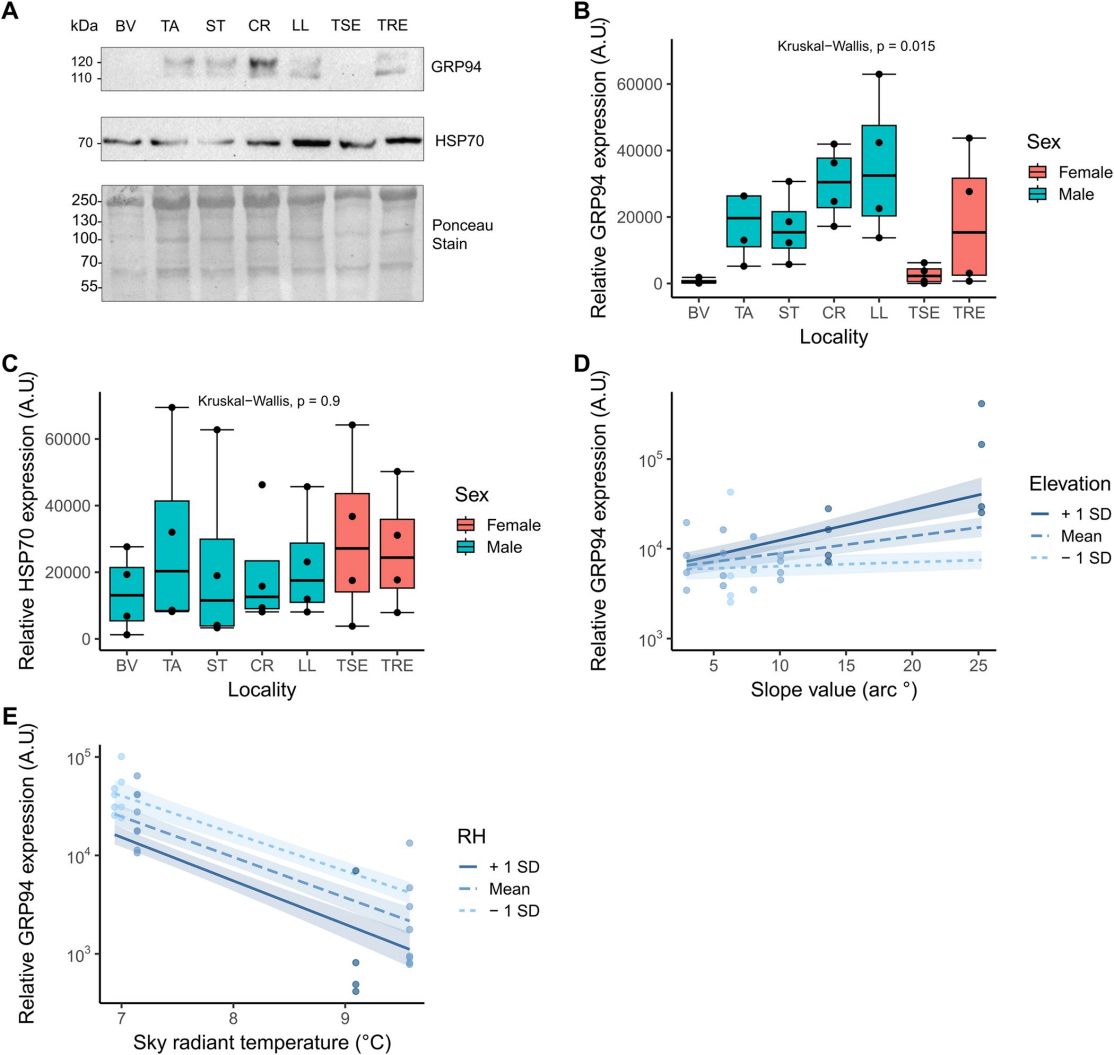
A) Representative western blot for HSPs with two bands appearing for GRP94, and one for HSP70. Molecular weights are labelled, and Ponceau S staining from the same membrane as a loading control is included in the lower panel. B) GRP94 and C) HSP70 expression measured in arbitrary units (A.U.) in *G*. *galloti* across different localities in Tenerife, from the lowest to the highest elevation. Sex is indicated by colour, where blue = males and red = females. D) and E) show significant interaction terms determined from model selection for GRP94 expression. GRP94 expression is plotted on the log_10_ scale. The main predictor is plotted on the x-axis and the moderating predictor, relative humidity (RH) is plotted as three separate lines, with a mean and ± 1 standard deviation (SD), with 50% confidence intervals. Points plotted are partial residuals which account for all variables in the model. The shade of the points corresponds to the moderator variable value.

The aim of this study was to shed light into the biochemical underpinnings of lizard physiological response to different environmental conditions. We first tested for local variation in the levels of GRP94 and HSP70, using western blot. GRP94 was typically identified as two protein bands appearing at 120 and 110 kDa, and HSP70 appeared as a single band at 70 kDa (Fig 2A and 2B). We found a general increase in median GRP94 expression with elevation, except for the two highest elevation sites, where samples showed reduced expression (Fig 2B). Samples from these two highest sites were from female animals only. There was a statistically significant difference in GRP94 levels between the localities (KW-H = 15.82, df = 6, p = 0.015), however, multiple Wilcox pairwise comparisons did not reveal significant differences between specific localities after Benjamini-Hochberg adjustment which accounts for multiple comparisons. Two-sample Wilcoxon tests showed no statistically significant differences between the two sexes (S4 Fig in S1 File). Median HSP70 expression did not change between the localities (KW-H = 2.19, df = 6, p = 0.90, Fig 2C).

We then used GLZs to describe our data. The best GLZ selected for GRP94 expression included two statistically significant interaction terms (where the effect of one variable on the outcome was different depending on the values of the other variable). These were slope angle (steepness), given elevation, and radiant sky temperature given relative humidity (Table 1). We found that GRP94 expression increased with site steepness (slope angle), and this effect was more prominent at higher elevations (Fig 2D). On the other hand, as sky radiant temperature increased, GRP94 expression decreased, with lower expression when interacting with higher relative humidity (Fig 2E). The GLZ for HSP70 expression data was an intercept-only model, meaning none of the predictors explained the observed expression (Table 1).

**Table 1:** Generalised linear models (GLZs) for the best set of predictors against the standardised expression of two HSPs and the two oxidative stress related PTM biomarkers. Model information includes all predictors, estimates, confidence intervals at 95% (CI), and p-value. Statistically significant predictors of ≤ 0.05 are shown in bold. The “:” indicated an interaction effect between two variables. “Slope” is the slope angle (measured in arc degrees°) and elevation is measured in m a.s.l. TSKYC refers to radiant sky temperature (°C) and RH refers to relative humidity (%). SOLR refers to solar radiation (W/m^2^) (unshaded, adjusted for slope, aspect, and horizon angle). General model information includes the degrees of freedom (DF), the R^2^ (Nagelkerke‘s pseudo R^2^ value), and AICc score. All models include all data points (n = 28 observations).

Biomarker	Predictor	Estimate	CI	p-value	DF	R^2^	AICc
GRP94	Intercept	16.3	14.5–18.0	**<0.001**	25	0.74	583.75
	Slope: Elevation	0.00003	0.000044–0.000016	**<0.001**			
	TSKYC: RH	-0.019	-0.023 –- 0.014	**<0.001**			
Carbonylation	Intercept	25.0	10.3–41.0	**<0.001**	24	0.34	592.93
	Sex	-7.11	-13.3 –-1.33	**0.013**			
	TSKYC	-0.90	-2.0–0.123	0.076			
	SOLR: Elevation	-0.00001	-0.000012–-0.000082	**0.013**			
HSP70	Intercept	10.07	9.7–10.4	**<0.001**	28	<0.001	623.13
3-NT	Intercept	15105.1	12975.6–17234.6	**<0.001**	28	<0.001	565.14

### 3.2 Levels of protein carbonylation, but not of 3-NT, vary with environmental factors

**Fig 3:**
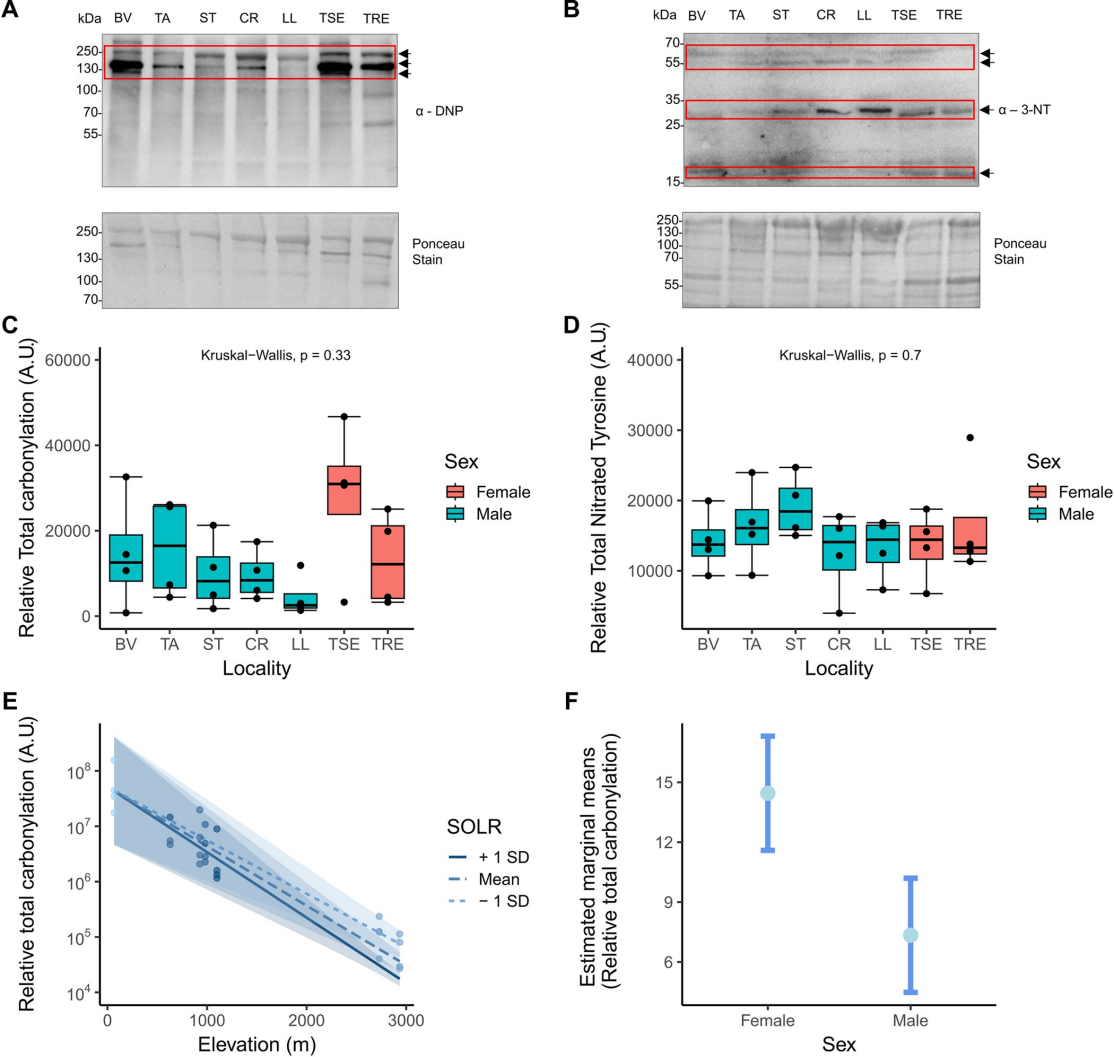
Representative Western blots (one of four biological replicates) for total carbonylation (A) and total 3-NT (B), labelled with molecular weights, and corresponding Ponceau S stain used as a loading control in the lower panel. Arrows and red boxes represent the bands quantified and the antibodies are labelled (α-DNP for the carbonylation assay and α-3-NT for 3-NT). C) Total carbonylation and D) 3-NT levels measured in arbitrary units (A.U) in *G*. *galloti* across different localities in Tenerife, from lowest to highest elevation. Sex is indicated by colour, where blue = males and red = females. E) Significant interaction terms determined from model selection for carbonylation, plotted on the log_10_ scale. Elevation is plotted on the x-axis and the moderating predictor (solar radiation) is plotted as three separate lines, with a mean and ± 1 standard deviation (SD), with 50% confidence intervals. Points plotted are partial residuals which account for all variables in the model. The shade of the points corresponds to the moderator variable value. F) Estimated marginal means between sexes for total carbonylation, where the central light blue point indicates the estimated marginal mean, and the error bars represent the standard error of estimate.

Next, to test for local variation in protein post-translational modifications associated with oxidative stress, we compared the standardised levels of protein carbonylation and 3-NT across localities (Fig 3A and 3B). Total carbonylation appeared to decrease slightly in median values with elevation, with exception of samples from the highest sites (taken from female animals) which were elevated, (Fig 3C), although this apparent decrease was not statistically significant (KW-H = 6.92, df = 6, p = 0.330). Total 3-NT remained constant across all localities (K-W = 3.83, df = 6, p = 0.700).

The selected GLZ for total carbonylation revealed aspect and slope-corrected solar radiation given elevation, and sex as statistically significant predictors (*post-hoc emmeans* table in S1 and S2 Tables in S1 File) (Table 1). Sky radiant temperature was also included in the final model, which showed a trend towards statistical significance (Table 1). The significant interaction term between elevation and solar radiation showed a decrease in total carbonylation with ascending elevation, at all levels of solar radiation (W/m^2^), which is unshaded, and adjusted for slope, aspect, and horizon angle (Fig 3E). Therefore, while solar radiation generally increases with elevation, in the case of our SOLR variable this was counteracted by steep sites receiving less radiation at the microclimatic scale, with vegetation also playing a role [71]. *Emmeans Post-hoc* analysis of the categorical variable “sex” as predictor within the total carbonylation model showed Tukey-adjusted estimated marginal means were higher in females than in males (Fig 3F). The final model selected for 3-NT was an intercept-only model, therefore none of the variables included were relevant to explain any variation in levels of total 3-NT.

### 3.3 Cross-talk between levels of carbonylation and GRP94

**Fig 4:**
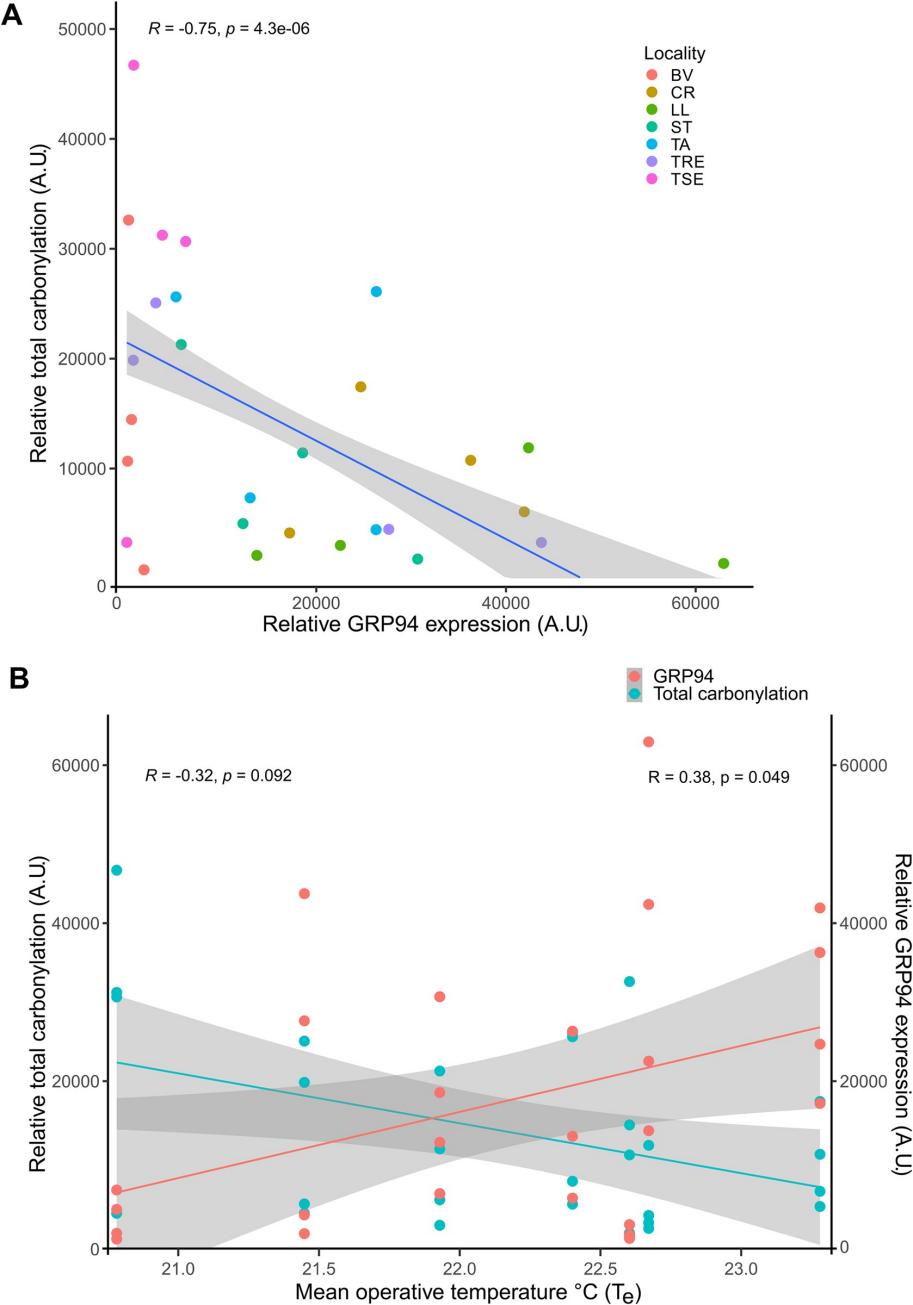
A) Negative correlation between relative total carbonylation and relative GRP94 expression with a linear fit and a 95% confidence interval. Raw observations are plotted with colours referring to localities Spearman correlation coefficient and p-value are calculated. B) Total carbonylation and GRP94 expression plotted against modelled mean (24 h) operative temperature°C (T_e_), Observed values for each protein are plotted and coloured, and linear fit show 95% confidence intervals. R correlation coefficient and p-value calculated from Spearman’s correlation coefficient for total carbonylation (left) and GRP94 (right).

Next, we tested the hypothesis that GRP94 expression was inversely correlated with levels of protein carbonylation. We found a statistically strong negative association (R = -0.75, p < 0.001), where total carbonylation was high when GRP94 expression was low, and *vice versa*, when plotted as a linear fit (Fig 4A). We then explored whether T_e_ on the day of collection could help explain the respective levels of carbonylation and GRP94. We found a trend towards decreased carbonylation at higher T_e_, and increased carbonylation at lower T_e_, (R = -0.32, p = 0.092). Contrarily, GRP94 was elevated at higher T_e_ and decreased with lower T_e_ (R = -0.38, p = 0.049) mirroring the relationship seen in Fig 4A. These results, when taken together, suggest that a delicate interplay between protein homeostasis (through GRP94 expression levels) and oxidative stress load (protein carbonylation levels) relate to available microclimates, with T_e_ values demonstrating lizards occupying different habitats face different challenges in balancing GRP94 and oxidative damage.

## 4. Discussion

The aim of this study was to shed light into the biochemical underpinnings of lizard physiological response to different environmental conditions. Ecological and phenotypic variation in *G*. *galloti* across Tenerife is well-studied and provided several examples of local adaptation across the island’s diverse microhabitats [61, 68, 85–90] When combined with phylogenetic studies detailing relatedness of populations on the island [66, 67, 91–93], a picture begins to emerge of how this species has evolved to cope with the varying environments on Tenerife. Despite this, a gap in knowledge remains regarding the molecular underpinnings of *G*. *galloti*’s adaptability.

Our results showed that the increase in GRP94 expression was determined by steeper slopes in the landscape, given higher elevations, and lower sky radiant temperature given lower relative humidity. Wind, drainage (leading to unsuitable vegetation and subsequent food sources associated with it [94]), sun exposure (with a -0.84 collinearity score against zenith angle), and shelter site availability may all be affected by the steepness of a site and are important microhabitat parameters for lizards [95]. Sky radiant temperature describes the amount of radiation emitted from the atmosphere, which subsequently affects organisms on the surface [96]. Sky radiant temperature is determined by particles in the air and atmosphere, including water, on which it can scatter and be re-emitted, altering the amount of radiation reaching the surface [97]. Solar radiation can induce HSP expression to protect cells *via* its role in the protein folding response [98]. Decreased GRP94 expression with higher sky radiant temperature and lower humidity may put lizards experiencing these conditions at risk of cellular stress, which is supported by the observation of higher protein carbonylation in localities with low GRP94 expression. GRP94 is upregulated in response to ultraviolet (UV) irradiation and plays a key role in mammalian melanogenesis–a key protective mechanism from solar activity [99]. Its role in squamate melanogenesis is not studied, however, lizards tend to be darker at higher elevations [100–102], where it helps thermoregulation and protects against UV radiation [103, 104], and *G*. *galloti* itself has black coloration [105]. This is consistent with a trade-off between the metabolic cost of maintaining higher GRP94 expression and accumulating oxidative damage. Solar radiation and elevation have previously been described as pertinent variables regarding thermal stress in ectotherms, with greater exposure leading to more stress events [106]. Complex combinations of environmental stressors can be matched with equally complex physiological cascades, including heat shock proteins, to promote organism survival [107, 108]. If stronger radiative variables can cause cellular damage and protein degradation within more complex landscapes, a response from molecular chaperones repair mechanisms will be elicited, thus explaining the relationship in our data.

The decrease in total carbonylation levels was determined by the interaction of solar radiation with elevation and by sex. The gradient of the solar radiation modulator was unexpected, since it typically increases at high elevations. However, solar radiation at the micro level is corrected by topography and obstructing vegetation, as previously mentioned [71]. Regarding sex, females can be more sensitive to oxidative damage than males in unfavourable environments, with subsequent effects on their immune system [90, 109–111]. Our results show that this is specific for protein carbonylation because there were no differences in the levels of 3-NT, which is associated with different oxidative stress pathways [112]. Females could be redirecting resources from maintaining constitutive GRP94 which, being glucose-regulated, comes at a metabolic cost. Protein expression can evolve to be “optimal” [4], therefore if resources are scarce such as at higher elevations there may be a cost to maintain constituent GRP94 [113–115]. This increases reproductive females vulnerability to extreme weather events in already stressful environments with limited resources [90, 116], as females may be unable to maintain GRP94 levels to a satisfactory extent to protect them against oxidative stress and protein carbonylation when supporting developing embryos [117]. Future research with a balanced design of males and females across localities is warranted for a more detailed assessment of such factors. The pattern of protein carbonylation observed here in a North-Western transect, matches that observed in *G*. *galloti* males in Southern elevational transects of *G*. *galloti* [68]. There, lowest total carbonylation was likewise observed in high elevation populations. Further support to this pattern comes from and another lacertid species (*Psammodromus algirus)*, which exhibited levels of oxidative stress decreasing at higher elevation [118] and higher elevations (with higher UV radiation) not inducing significantly more oxidative damage or an antioxidant response [119]. A high threshold level of solar radiation may be required for oxidative stress to occur, and that may also depend on other stressors [120].

A clear inverse relationship was found between GRP94 and carbonylation levels. Molecular chaperones or stress proteins can prevent protein carbonylation and cell death through calcium homeostasis [22, 121, 122]. In fish, it was demonstrated that HSPs directly protect against oxidative stress in natural populations [34, 123], highlighting their functional role in protein folding at both constitutive and inducible levels for organisms in stressful environments. [23] presented experimental evidence in mouse tissue of how GRP94 specifically plays a foundational role in protection against, and active reduction of oxidative stress (carbonylation) biomarkers in muscle tissues, highlighting its role as a cytoprotective agent. The role of GRP94 as a member of the HSP90 family is well characterised [20, 21]. However this is the first study to our knowledge linking its cytoprotective abilities with lower total carbonylation levels in natural populations of lizards. Our results demonstrate that molecular chaperones such as GRP94 not only play a role in the restoration of cellular homeostasis through its role as a protective agent, but also interact with evolutionary processes. These molecules can also act as a buffer to selection through their role in cellular stabilisation, which in turn can regulate the release of genetic variants [6, 124–126], impacting the evolvability of traits involving these molecules. The onset of expression of these markers should also be considered when studying acute and chronic stressors. Because we sampled lizards from wild populations, we must consider that they may have experienced different environmental stressors at different timescales, even within the same locality. Indeed, *G*. *galloti* may live up to 11 years in the populations analysed [68]. This may manifest as differences in the age and composition of studied tissues, depending on the longevity of markers following more acute stressors.

While HSP70 has been found to increase following heat stress in other lizard species [127–130], the uniform expression of HSP70 observed in this study can be explained by the lack of an acute cellular stress stimulus [131, 132], which aligns with reports that HSP70 baseline expression without being subjected to heat-shock experiments can be constant [127, 129, 133, 134].

By modelling mean T_e_, we estimated average temperatures of an inanimate ‘lizard’, with zero heat capacity exposed to the same microclimate, which is regarded as a meaningful proxy of thermoregulatory opportunities [78, 135]. This was done for each population, over a 24 hour period. T_e_ does not account for behavioural thermoregulation and is therefore a distinct measurement from body temperature. Other metabolic regulators such as glucocorticoids have been shown to vary with T_e_, [136]. However, it is important to acknowledge the potential for long-term stability or baseline fluctuations in these biomarkers, which could be influenced by various factors such as daily and seasonal variation, or reproductive status, for which we currently lack information. As the T_e_ model includes data from all localities, it does not consider intraspecific differences in thermal preference and water loss between localities or subspecies [68], but serves as a guiding principle of the relationship between experienced T_e_ and biomarkers. The modelled values of T_e_ suggest an equilibrium point (at 21.9°C in our model, but note section in Methods on the interpretation of this parameter) around which lizards may undergo a trade-off between the metabolically costly maintenance of GRP94 at higher mean T_e_ to lower oxidative cell damage, and decreased metabolic rate at lower mean T_e_ through lower GRP94 baseline expression, leading to accumulation of oxidative damage [9]. Given the narrow thermal range either side of the equilibrium point, this gives importance to behavioural thermoregulation which can help keep the balance between these alternatives. Thermoregulatory behaviour may also be driven by factors other than energy expenditure [63], and the overall cost of oxidative stress and cellular protection is not well quantified. Despite this, there is strong evidence that lizards experiencing high temperatures vastly exceed their metabolic capacity [137]. Hydration is also an important factor to consider influencing thermoregulation [138, 139] which was not included in T_e_ modelling. However, relative humidity is considered in the GLZ as a significant predictor for GRP94 expression, interacting with sky radiative temperature, supporting the salience of hydric, as well as thermal pressures in *G*. *galloti* [90, 140, 141].

## 5. Conclusions

Molecular chaperones can exhibit plastic patterns in the physiological and metabolic response to environmental stressors [142], impacting evolutionary processes with respect to the environment [143]. The differences in constitutive biomarker expression across *G*. *galloti* populations (from the same phylogenetic lineage and independently of subspecific status) here may be an example for such plastic responses, and contribute to “frontloading”, “preparation for oxidative stress” or “pre-adaptation” [7–10]. GRP94 may even have survival consequences due to its involvement in melanogenesis, [99], with darker individuals better able to thermoregulate given levels of solar radiation [144]. Further investigation is required to explore the relationship between thermal-adapted phenotypes and behaviours, with molecular chaperones and cellular stress, and if there is an impact on evolutionary processes or population dynamics. The role of molecular chaperones are key in addressing adaptive capacity not just at the physiological level, but at the evolutionary level [6, 145]. These plastic responses we observed here may incur a cost, leading to equilibrium dynamics around energy use vs. cellular protection trade-offs at different operative temperatures. Our findings not only highlight GRP94 and carbonylation as useful biomarkers when assessing cellular stress and protection in wild populations, but also demonstrate that thermoregulatory behaviours (through operative temperature) provided by a range of microclimates are critical in maintaining a balanced internal biochemistry. This may assist in conservation of vulnerable lizards when measuring individual health and managing habitats.

## Supporting information

S1 File Supplementary methods, five supplementary figures, four supplementary tables, supplementary references. (DOCX)

S1 Raw images Original, raw western blot images. (DOCX)

## Abbreviations

PTM: Post translational modification
HSP: Heat shock protein
3-NT: 3-Nitrotyrosine
ER: Endoplasmic reticulum
T_e_: operative temperature
TBST–: Tris buffered saline with Tween 20
SDS-PAGE: Sodium dodecyl sulfate-polyacrylamide gel electrophoresis
PVDF: Polyvinylidene difluoride
BSA: Bovine serum albumin
HRP: Horseradish peroxidase
DNP: Dinitropheny
DEM: Digital elevation model
AICc: Akaike information criterion corrected
GLZ: Generalised linear model
