# Supplementary material for: Environmental factors influence cross-talk between a heat shock protein and an oxidative stress protein modification in the lizard Gallotia galloti
Source: PLoS One. 2024 Mar 12;19(3):e0300111. doi: 10.1371/journal.pone.0300111 (PMC10931494; doi:10.1371/journal.pone.0300111)
Supplement: S1 File — (DOCX) [file pone.0300111.s001.docx]

**Supplementary materials**

to the manuscript

**Environmental factors influence cross-talk between a heat shock protein and an oxidative stress protein modification in the lizard *Gallotia galloti***

**Contains:**

- **Supplementary Material 1. Methods**
- **5 Supplementary Figures**
- **4 Supplementary Tables**
- **Supplementary References**

**Supplementary Material 1. Methods**

**Protein extraction and antibody testing**

To produce protein lysates for western blotting, between 20 mg and 300 mg of tail tip tissue (depending on size and amount of starting tissue) were immersed in liquid nitrogen and ground into a powder. Combined with lysis buffer (2% SDS in 50 mM Tris, 150 mM NaCl) at a ratio of 1:10, tissue was lysed with a high-powered electric homogenizer (Ultra-Turrax T25). The lysate was incubated at 4 °C for 1 h, then centrifuged for 15 min at 4,000 rpm, transferring the protein supernatant to a new tube. Protein concentrations were quantified from the lysate through a Pierce BCA protein assay (ThermoFisher Scientific, 23225),and all samples were then normalised to a protein concentration of 1 µg/µl. Laemmli Buffer was added to each sample in a 1:4 ratio before being loaded into gels for SDS-PAGE.  To test the cross reactivity of antibodies (which are often not designed for squamate reptiles), we trialled the performance of antibodies GRP94 (cell signalling, #2104), HSP70 (Monoclonal (3A3) Invitrogen, #MA3-006) and the protein carbonyl assay (ab178020), on existing reptile tail tips from preserved tissue of the gecko species *Hemidactylus turcicus,* and used this as a positive control for standardisation purposes on all membranes. Samples were boiled at 100 °C for 10 min to denature proteins before loading. Twenty µl of sample and 10 µl of ladder (Thermo Sci PageRuler, 11852124) were loaded in 12% acrylamide gels for SDS-PAGE. The gel was run for approximately 1.5 h at 120 V and proteins were transferred onto a nitrocellulose or PVDF membrane for 1 h at 100 V. The membranes were then blocked in a solution of 5% milk/TBST for 1 h at room temperature. The membranes were washed three times in TBST solution for 10 min each, before being incubated with the primary antibody in a 5% milk/TBST solution at a 1:1,000 dilution or 1:500 dilution for GRP94, overnight. The membrane was washed as before, and then incubated with secondary antibodies.. After washing, chemiluminescence was visualised (#WBLUF0500, Millipore) on a Bio-Rad Chemidoc. Exposure times varied depending on the protein and the membrane and were subsequently corrected for during protein band standardisation (Bass et al., 2017).

**Band densitometry and standardisation**

To standardise protein bands from each Western blot image, the total protein density (from Ponceau staining) was measured in ImageJ (Schneider et al., 2012) for each lane, to determine a value. A lane standardisation factor was calculated using the ratio of each lane value and the highest lane value on that membrane (Bass et al., 2017). Target protein band area can then be divided by that lane standardisation factor.

Other standardisation calculations were performed, including relative to the positive control sample “gecko tail” for which identical lysate was used on each of the membranes, and values relative to the housekeeping protein GAPDH.

All standardisation methods demonstrated the same relationships between biomarkers and localities and did not deviate from each other in preliminary data exploration. However, quantifying western blot bands as a proportion of total protein density avoids potential bias that can occur using measurement of housekeeping proteins (e.g., GAPDH) (Bass et al., 2017). Factorisation to total protein density had the lowest mean absolute deviation for the positive control “gecko tail” across membranes, therefore the “total protein factor” standardisation method was used moving forward for downstream analysis (Supplementary, Figure 1).

All bands present for the two candidate proteins, GRP94 and HSP70 were quantified, and protein carbonylation was investigated at 250kDa, 130kDa, and 120kDa molecular weights, as well as the sum of these bands (described as “total carbonylation”). 3-NT bands were investigated at approximately 65kDa, 55kDa, 30kDa, and 18kDa, as well as the sum of these bands (“total nitrated tyrosine”).

**Microclimate during the sampling period**

A repeated measures ANOVA was conducted to test for temporal differences between varying timepoints for each of the variables modelled from *microclim*, to demonstrate that daily data modelled on the day of sampling is representative and within the timeframe of maximum molecular chaperone expression. Timepoints representing before, during, and after the day of sampling are analysed. Significant differences were found for solar radiation between before and after sampling (timepoint 1 and 3), but not during sampling (timepoint 2). Radiant sky temperature showed significant differences before and after, and during and after. This showed that while these variables may have large degrees of variability on the temporal scale, there were no significant differences between the days preceding sampling and the day of sampling, which influenced biomarker expression, therefore justifying using data on the day of sampling.

In addition, tail tips were sampled during, 1 day after, and 5 days after field collection, due to inclusion in other experiments. To account for this, processing time was included as a predictor variable. Despite being collinear with other variables which were not highly ranked in model selection (Figure S2), we confirmed it had no influence over biomarker expression by including it in the final model selection stage, where it did not appear as an included term for any of the models.

**Supplementary Figures**


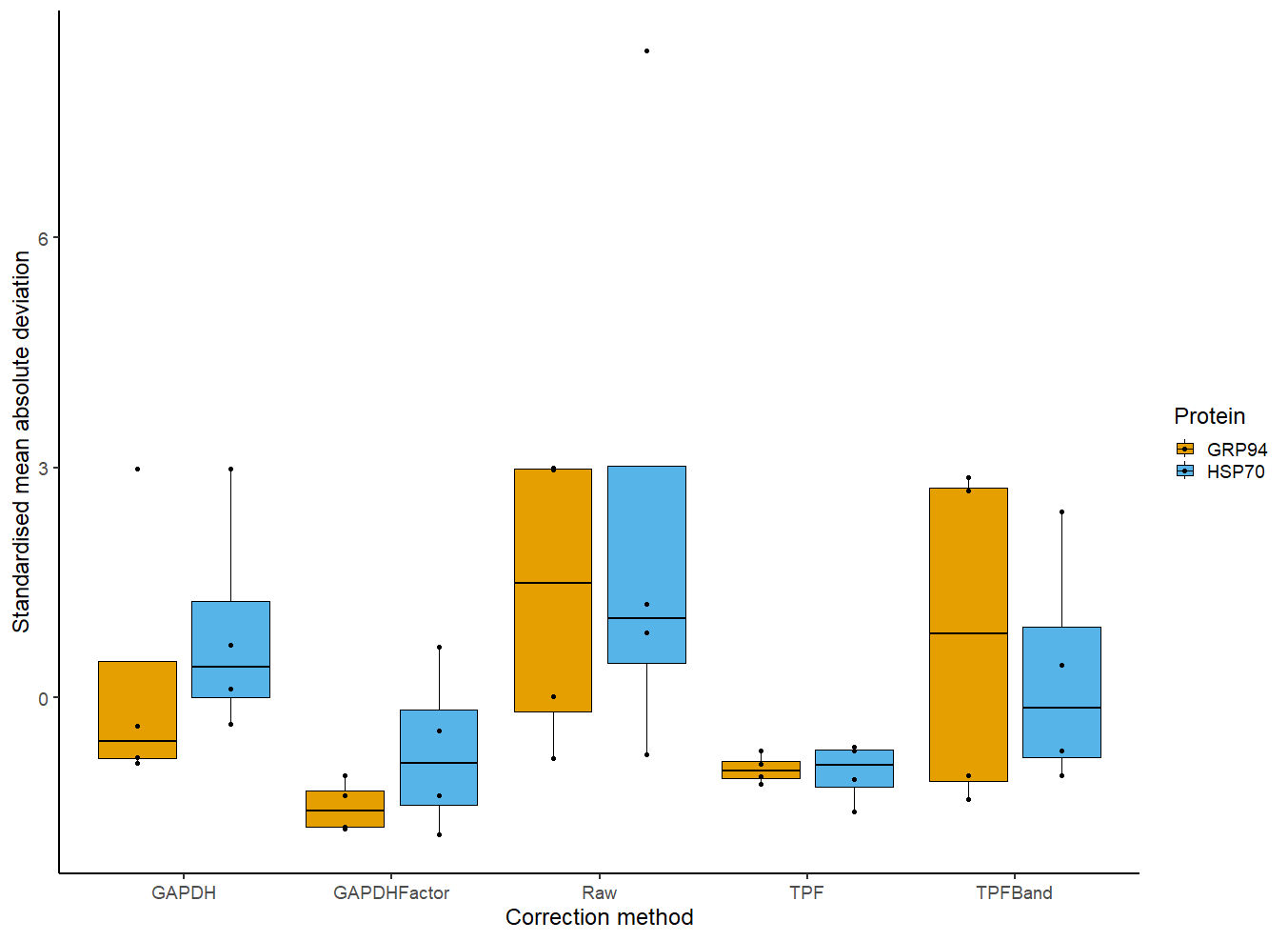


**Supplementary Figure S1**. Standardised absolute deviations from the mean across all membranes of transformed densitometry values of the positive control for GRP94 and HSP70. X axis categories represent the different correction methods. Since the same lysate was used across all membranes, the correction method producing the lowest absolute deviation values across proteins and membranes should be the preferred method. GAPDH - ratio of GAPDH; GAPDHfactor - ratio of GAPDH factor; Raw – raw densitometry values; TPF - ratio of total Protein Factor (Ponceau Stain); TPFBand - ratio of Total protein factor from a single band (Ponceau Stain).


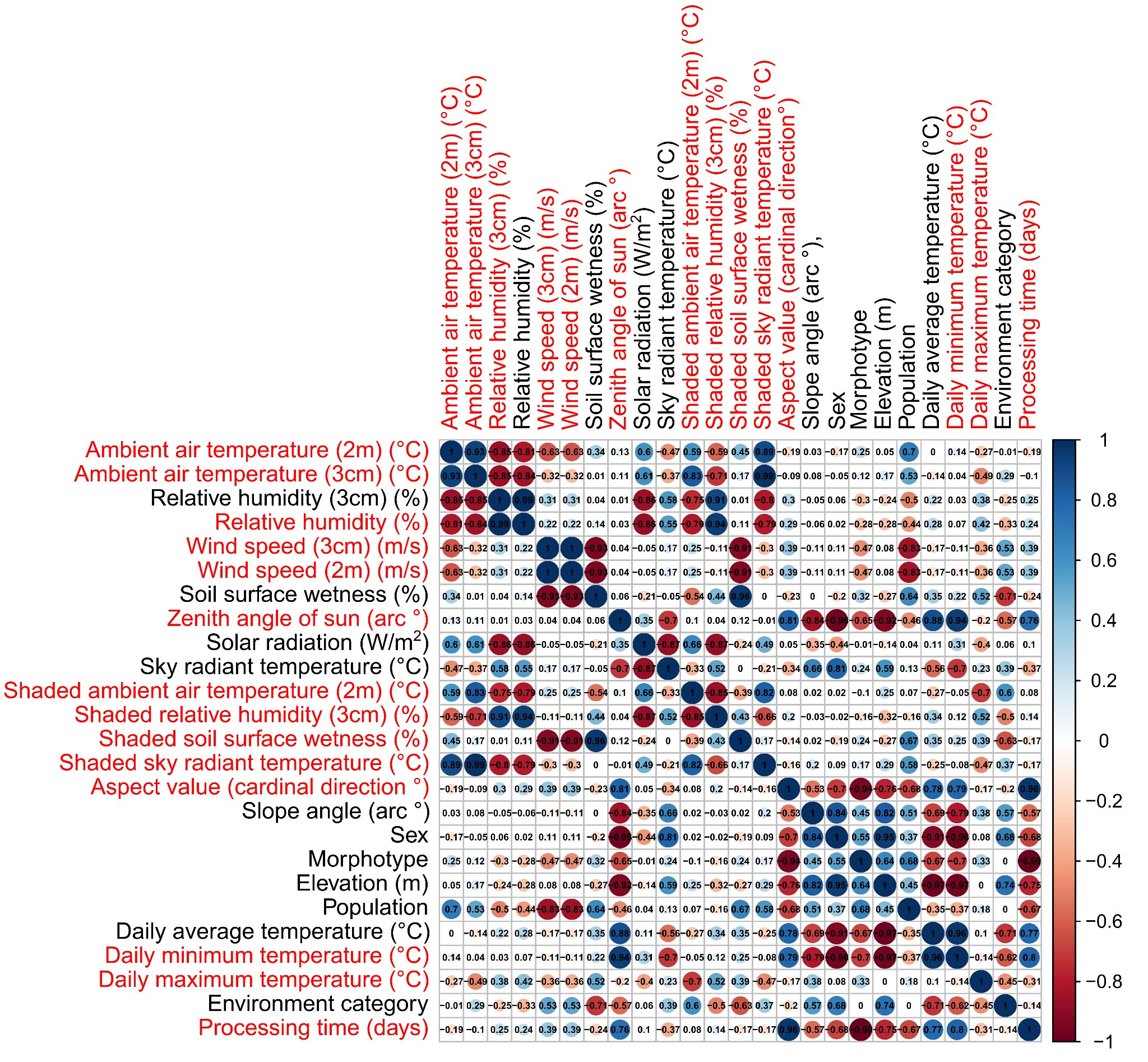


**Supplementary Figure S2**. Collinearity plot showing all predictors assessed before removal prior to model selection at collinearity score >0.9. The numbers indicate collinearity score, the size of the circle indicates collinearity strength (analogous to the score), and the colour represents negative (red) or positive (blue) correlations. Variables in red text represent those subsequently removed.


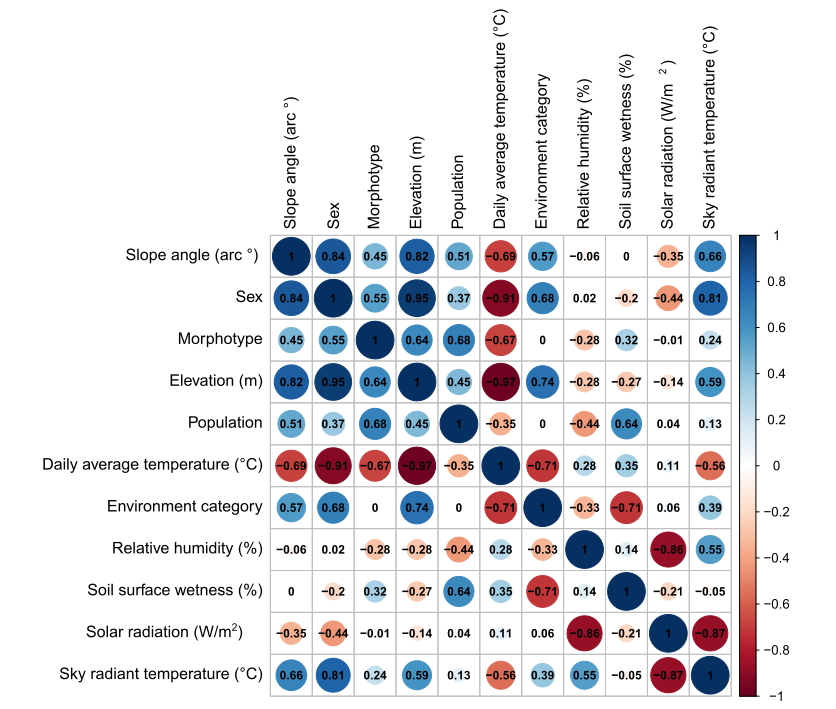


**Supplementary Figure S3**. Collinearity plot showing the final predictors included for model selection. The numbers indicate collinearity score, the size of the circle indicates collinearity strength (analogous to the score), and the colour represents negative (red) or positive (blue) correlation. Note that the variables: Sex, Elevation, and Daily average temperature were included for ease of interpretation despite a collinearity score of >0.9 with each other.


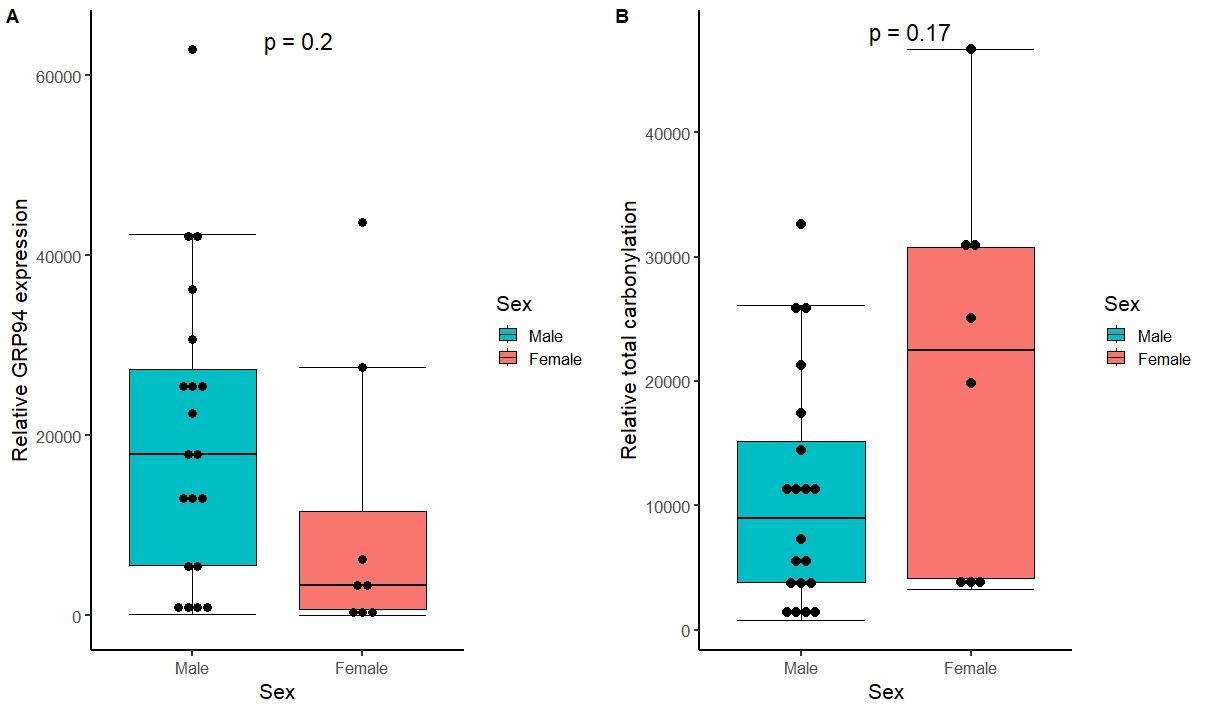


**Supplementary Figure S4**. Boxplots showing GRP94 expression (A) and total carbonylation (B) for male and female lizards. There are no statistically significant differences between the sexes for either biomarker (two-sample Wilcoxon test, A: W=106, p=0.199, B: W=52, p=0.165), although there seems to be a trend towards less relative GRP94 expression and more total carbonylation in females.


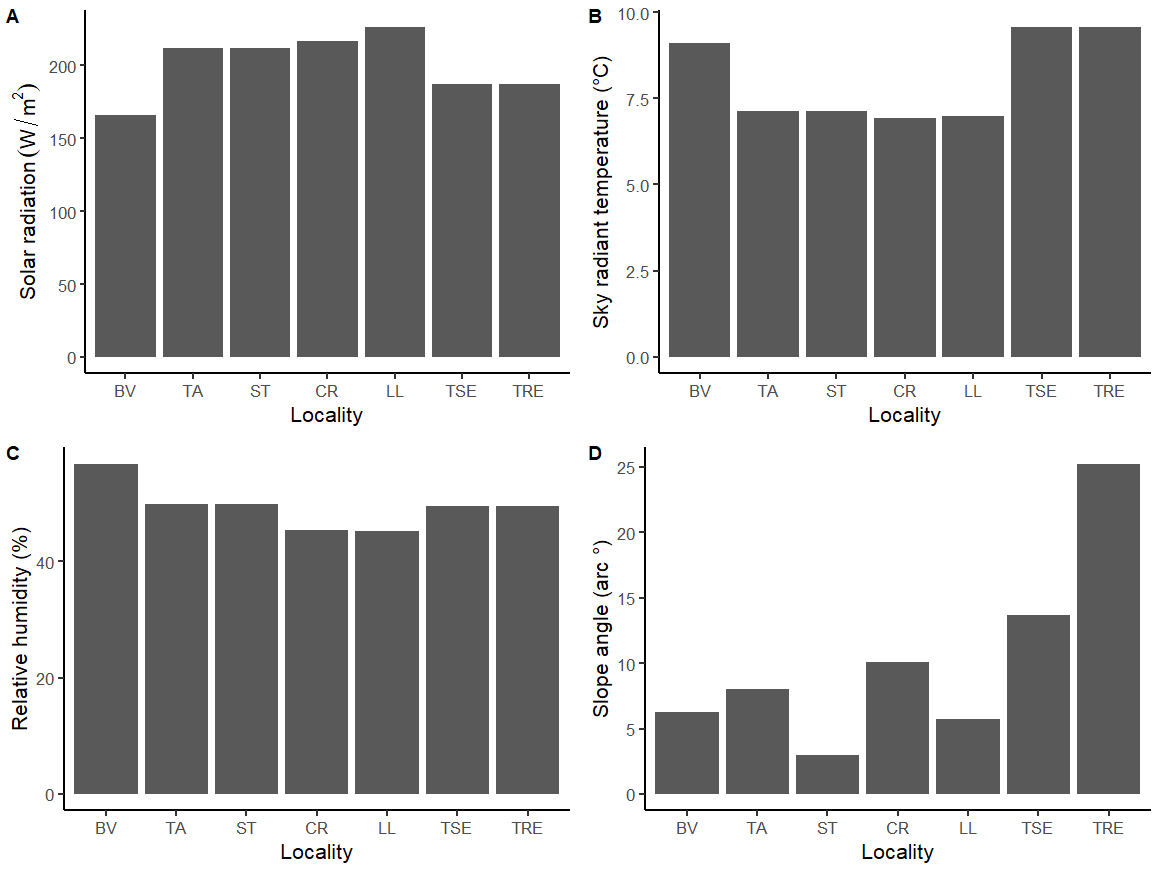


**Supplementary Figure S5**. Values of environmental variables for each locality in ascending elevation. A) Solar radiation (unshaded, adjusted for slope, aspect and horizon angle), B) sky radiant temperature (°C), C) relative humidity (%), and D) slope angle (arc °), or steepness. Of note is the non-linearity of solar radiation and sky radiant temperature with increasing elevation, which are markedly higher (B) or lower (A). Site abbreviations as follows, followed by the elevation in metres: Buena Vista (67) (BV), El Tanque (629) (TA), Santiago del Teide (927) (ST), Cruz de Tea (981) (CR), Los Llanos (1099) (LL), Bottom of the slope of Teide (2729) (TSE), and below the Altavista refuge, Teide (2935) (TRE).

**Supplementary Tables**

**Supplementary Table S1**. Post-hoc analysis of sex from the carbonylation model using the *emmeans* package (Lenth et al., 2019) in R. Values for both sexes are shown, with estimated marginal means (EMM), standard error (SE), degrees of freedom (DF), and lower and upper confidence limits (CL).

| Sex | EMM | SE | DF | Lower CL | Upper CL |
| --- | --- | --- | --- | --- | --- |
| Female | 14.5 | 2.1 | 24 | 10.2 | 18.8 |
| Male | 7.3 | 0.86 | 24 | 5.5 | 9.1 |

**Supplementary Table S2**. Contrast between sexes for carbonylation, with the estimate, standard error (SE), degrees of freedom (DF), t-ratio, and Tukey adjusted p-value. Statistical significance ≤0.05 are in bold.

| Contrast | Estimate | SE | DF | t-ratio | p-value |
| --- | --- | --- | --- | --- | --- |
| Female - Male | 7.2 | 2.9 | 24 | 2.5 | **0.020** |

**Supplementary Table S3**. Repeated measure ANOVA showing temporal difference in variables generated from *microclim* at 3 timepoints. Variables included are solar radiation *(*W/m^2^*)* (SOLR), radiant sky temperature (°C) (TSKYC), and relative humidity (%) (RH). Timepoint 1 = 2, 3, and 4 days before sampling, timepoint 2 = 1 day before, day of sampling, and day after sampling, timepoint 3 = 2, 3, and 4 days after sampling. Statistically significant differences of p < 0.05 are highlighted in bold.

| **Variable** | **Effect** | **DFn** | **DFd** | **F** | **P** | **Ges** |
| --- | --- | --- | --- | --- | --- | --- |
| SOLR | Site | 6 | 42 | 0.781 | 0.589 | 0.100 |
|  | Timepoint | 2 | 42 | 3.840 | **0.029** | 0.155 |
|  | Site:Timepoint | 12 | 42 | 0.383 | 0.962 | 0.099 |
| TSKYC | Site | 6 | 42 | 0.246 | 0.958 | 0.034 |
|  | Timepoint | 2 | 42 | 5.255 | **0.009** | 0.200 |
|  | Site:Timepoint | 12 | 42 | 0.287 | 0.988 | 0.076 |
| RH | Site | 6 | 42 | 0.445 | 0.845 | 0.060 |
|  | Timepoint | 2 | 42 | 1.679 | 0.199 | 0.074 |
|  | Site:Timepoint | 12 | 42 | 0.668 | 0.771 | 0.160 |

Su**pplementary Table S4.** *Post-hoc* pairwise comparisons between significant timepoints before and after field sampling from variables generated from *microclim*, including Holm-adjusted p-values. Variables included are solar radiation *(*W/m^2^*)* (SOLR) and radiant sky temperature (°C) (TSKYC). Statistically significant differences of p < 0.05 are in bold*.*

| **Y** | **Group 1** | **Group 2** | **N1** | **N2** | **Statistic** | **df** | **p** | **p.adj** |
| --- | --- | --- | --- | --- | --- | --- | --- | --- |
| SOLR | **Timepoint 1** | **Timepoint 2** | 21 | 21 | -0.671 | 20 | 0.51 | 0.51 |
|  | Timepoint 1 | Timepoint 3 | 21 | 21 | -2.84 | 20 | **0.01** | **0.03** |
|  | Timepoint 2 | Timepoint 3 | 21 | 21 | -2.12 | 20 | 0.047 | 0.094 |
| TSKYC | **Timepoint 1** | **Timepoint 2** | 21 | 21 | 0.235 | 20 | 0.816 | 0.816 |
|  | Timepoint 1 | Timepoint 3 | 21 | 21 | 3.69 | 20 | **0.001** | **0.004** |
|  | Timepoint 2 | Timepoint 3 | 21 | 21 | 2.97 | 20 | **0.008** | **0.015** |

**Supplementary References**

Bass, J. J., Wilkinson, D. J., Rankin, D., Phillips, B. E., Szewczyk, N. J., Smith, K. & Atherton, P. J. (2017) An overview of technical considerations for Western blotting applications to physiological research. *Scandinavian journal of medicine & science in sports*, 27(1), 4-25.

Lenth, R., Singmann, H., Love, J., Buerkner, P. & Herve, M. (2019) Package ‘emmeans’. *R package version*, 1(3.2).

Schneider, C. A., Rasband, W. S. & Eliceiri, K. W. (2012) NIH Image to ImageJ: 25 years of image analysis. *Nature methods*, 9(7), 671-675.
