# Supplementary material for: Environmental factors influence cross-talk between a heat shock protein and an oxidative stress protein modification in the lizard Gallotia galloti
Source: PLoS One. 2024 Mar 12;19(3):e0300111. doi: 10.1371/journal.pone.0300111 (PMC10931494; doi:10.1371/journal.pone.0300111)
Supplement: S1 Raw images — (DOCX) [file pone.0300111.s002.docx]

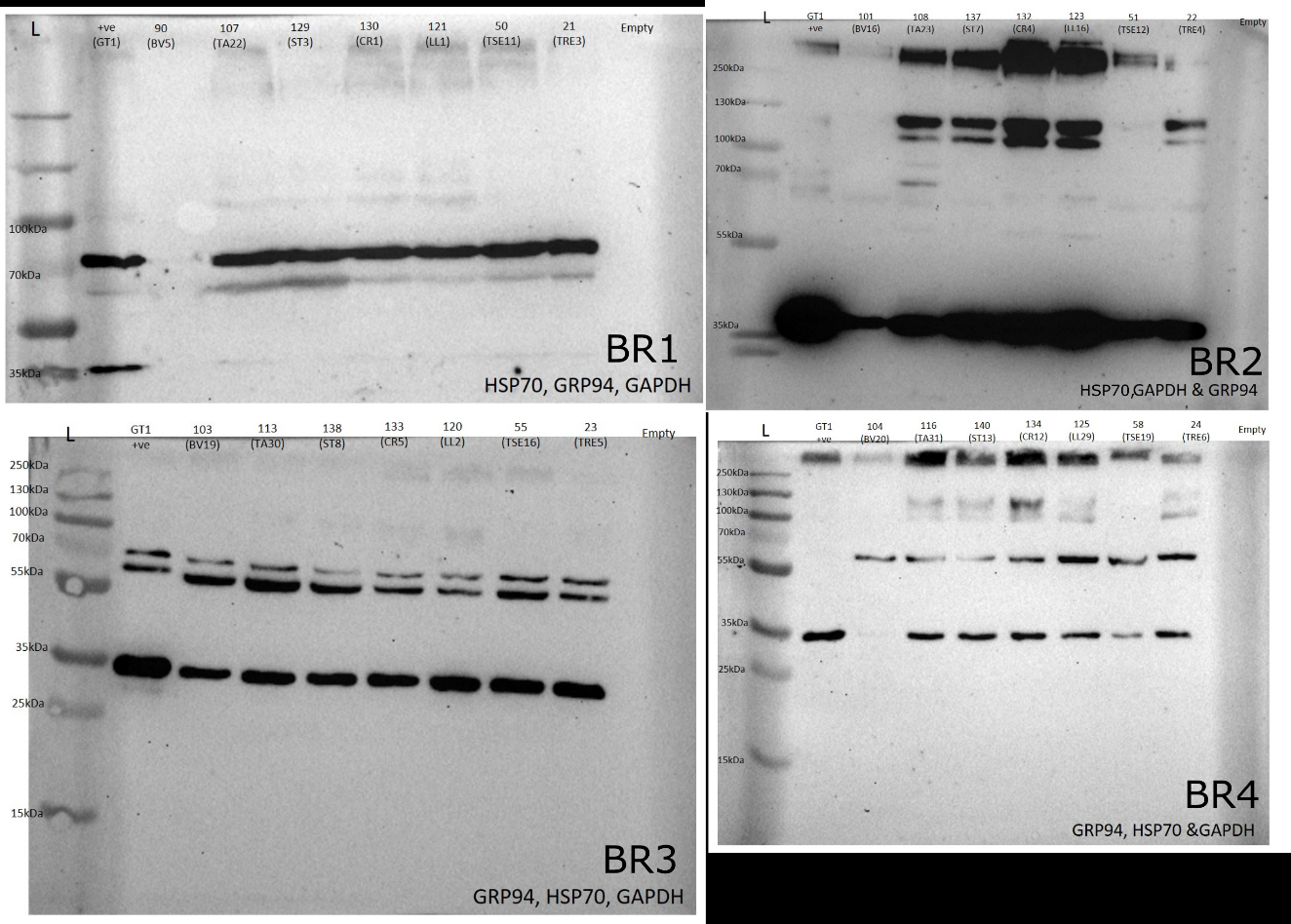


Figure 1. Original blot images for each set of biological replicates. Image captured with a ChemiDoc MP Imaging System. Each blot images shows the molecular ladder, a positive control (GT1), and samples for each locality. The last lane is empty. Each blot has been incubated with GRP94, HSP70, and GAPDH. GRP94 produced high molecular weight bands at > 250kDa, and two bands at 110 and 120kDa. HSP70 produced one or two bands at 70kDa. GAPDH produced bands at 35kDa as a control, however was not included in any analyses.


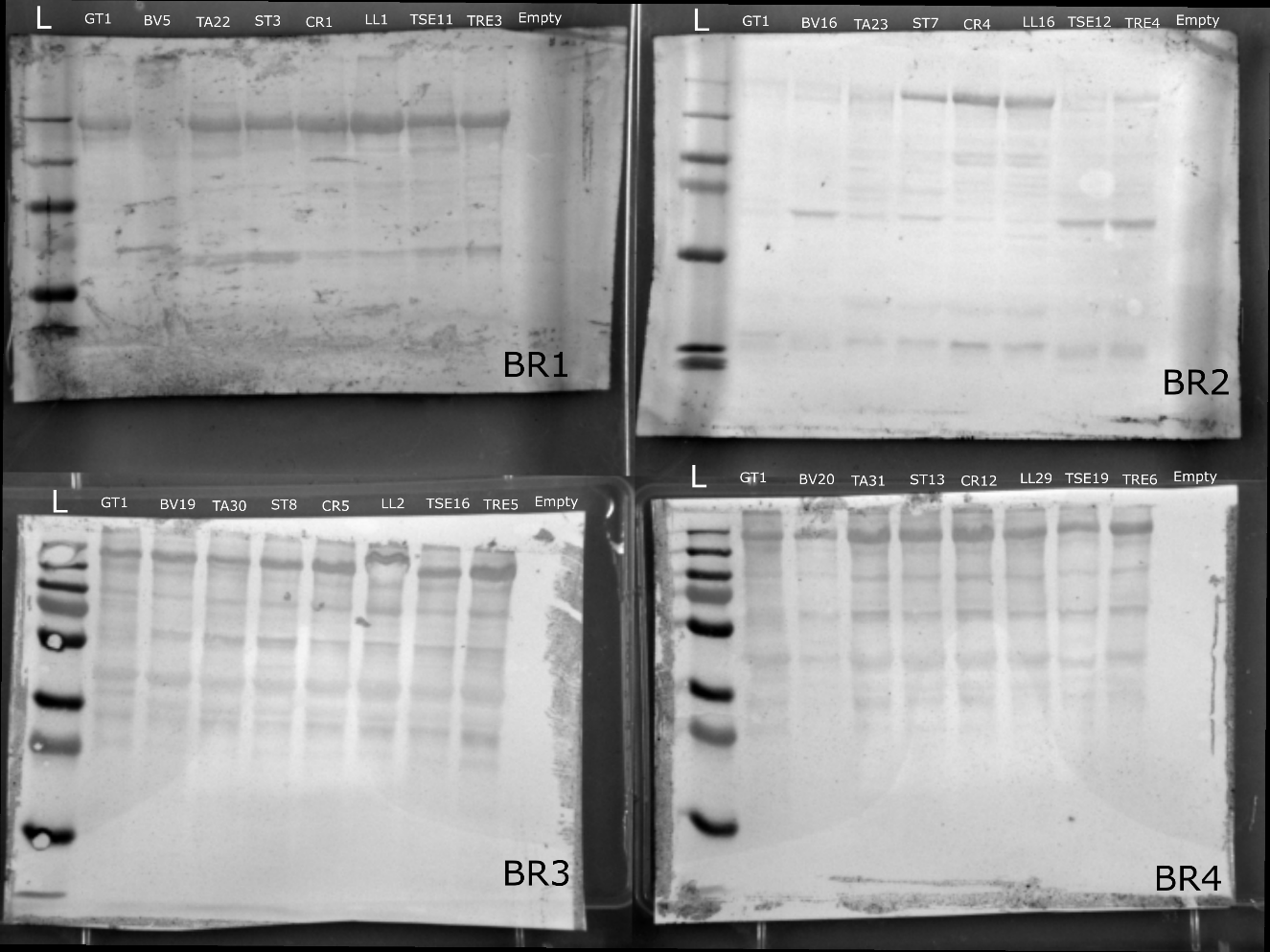


Figure 2. Ponceau stains from the same blots shown in Figure 1. Image captured with a ChemiDoc MP Imaging System. The Ponceau stain was used to quantify the band intensity relative to the total protein content loaded into the lane revealed by the Ponceau.


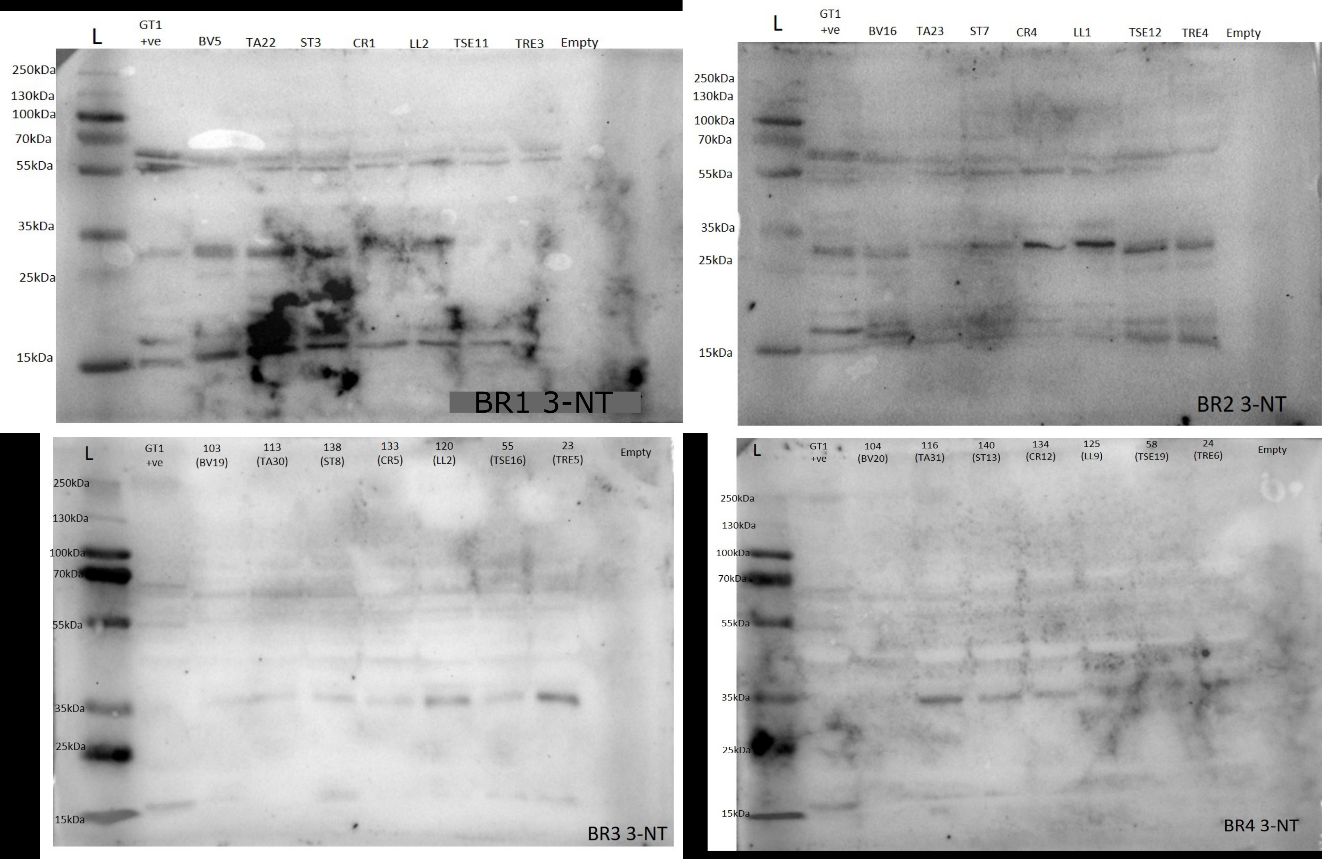


Figure 3. Blots of 3-Nitrotyrosine incubation using the same samples (sets of biological replicates). Image captured with a ChemiDoc MP Imaging System. Bands were quantified at the 65, 55, 30, and 18kDa.


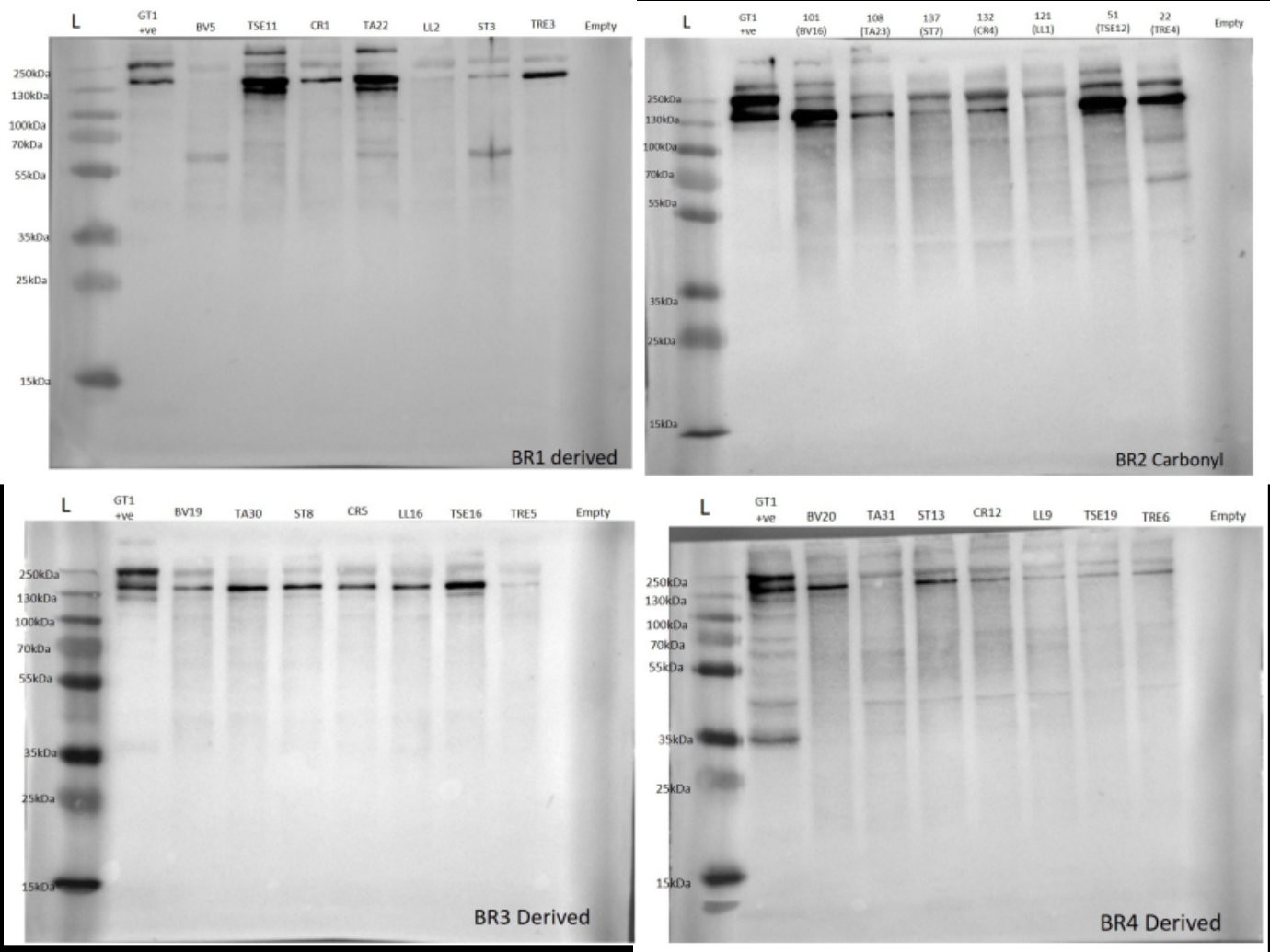


Figure 4. Blots of carbonylation incubation using the same samples (sets of biological replicates). Image captured with a ChemiDoc MP Imaging System. Bands were quantified at the 250, 130, and 120kDa.
